# Impact of Frailty on the Prognosis of Patients With Liver Cirrhosis Undergoing Insertion of a TIPS

**DOI:** 10.1111/apt.70315

**Published:** 2025-08-01

**Authors:** Martin Andreas Kabelitz, Simon Johannes Gairing, Anja Tiede, Eva Maria Schleicher, Liv Grete Ahl, Lea Wagner, Falko Zucker‐Reimann, Hannah Rieland, Jim Benjamin Mauz, Julia Weinmann‐Menke, Bernhard C. Meyer, Michael Bernhard Pitton, Heiner Wedemeyer, Peter Robert Galle, Lisa Sandmann, Benjamin Maasoumy, Christian Labenz

**Affiliations:** ^1^ Department for Gastroenterology, Hepatology, Infectious Diseases and Endocrinology Hannover Medical School Hannover Germany; ^2^ Department of Internal Medicine I University Medical Center of the Johannes Gutenberg‐University Mainz Germany; ^3^ Cirrhosis Center Mainz (CCM), University Medical Center of the Johannes Gutenberg‐University Mainz Germany; ^4^ German Center for Infectious Research, Partner Site Hannover‐Braunschweig Braunschweig Germany; ^5^ Department of Diagnostic and Interventional Radiology Hannover Medical School Hannover Germany

**Keywords:** frailty, hepatic encephalopathy, liver cirrhosis, TIPS, transjugular intrahepatic portosystemic shunt

## Abstract

**Background and Aims:**

Frailty is associated with a poorer prognosis of patients awaiting liver transplantation. Data on the impact of frailty on prognosis after transjugular intrahepatic portosystemic shunt (TIPS)‐insertion in patients with cirrhosis and the influence of TIPS on longitudinal changes in frailty are lacking.

**Methods:**

We retrospectively analysed data of 123 prospectively recruited patients with cirrhosis in Mainz and Hannover prior to elective TIPS insertion and monitored them for death/liver transplantation or post‐TIPS overt hepatic encephalopathy (OHE). Patients underwent testing with the Liver Frailty Index (LFI) prior to TIPS insertion as well as 1, 3 and 6 months after TIPS placement.

**Results:**

Median LFI prior to TIPS insertion was 4.32 (interquartile range: 3.78–4.88). 53% of patients who were frail at baseline and still alive at 6 months improved to prefrail status within 6 months of TIPS insertion. Higher LFI and younger age were associated with a decrease in LFI within 6 months. During follow‐up, 40 patients developed post‐TIPS OHE and 30 patients died or received a liver transplantation. There was no significant association between LFI as a metric variable and post‐TIPS OHE or liver transplantation/death. However, patients with LFI values in the lowest quartile had a significantly better transplantation‐free survival.

**Conclusions:**

TIPS insertion seems to improve physical functioning, as indicated by a decreasing LFI, but only in patients with a poor performance in LFI prior to TIPS. Conducting LFI prior to elective TIPS insertion can identify those with an excellent prognosis. However, frailty should not be considered a contraindication for TIPS.

**Trial Registration:**

ClinicalTrials.gov identifier: NCT05466669 and NCT04801290

## Graphical abstract

In patients with cirrhosis undergoing TIPS, frailty—measured by the Liver Frailty Index—improved in those with poor baseline status; therefore, high frailty should not be seen as a contraindication to elective TIPS. Additionally, LFI assessment before TIPS enables identification of patients with excellent survival prognosis.

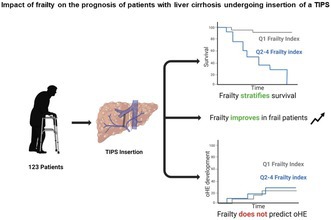

**Abbreviations**
ALD: alcoholic liver disease
ANT: animal naming test
FIPS: Freiburg index for post‐TIPS survival
LFI: liver frailty index
MASLD: metabolic dysfunction‐associated steatotic liver disease
MELD: model for end‐stage liver disease
OHE: overt hepatic encephalopathy
PHES: psychometric hepatic encephalopathy score
TIPS: transjugular intrahepatic portosystemic shunt

## 1 Introduction

Liver cirrhosis is among the most common causes of death worldwide [1]. In Germany, hospitalisations due to complications of cirrhosis are increasing [2]. Portal hypertension is one of the main pathophysiological triggers for most complications of cirrhosis and can be effectively treated by insertion of a transjugular intrahepatic portosystemic shunt (TIPS) [3, 4]. TIPS has been proven to be highly efficient for reducing the risk of re‐bleeding in patients with a history of variceal haemorrhage and for controlling ascites.

In this context, a recent meta‐analysis even demonstrated a survival benefit for TIPS in patients with ascites [5]. However, TIPS does not improve prognosis in every treated patient, and identifying patients without a treatment benefit in clinical practice remains challenging. Decades ago, the model for end‐stage liver disease (MELD) was developed to predict survival in patients with TIPS and still remains one of the mainstays for risk stratification [6]. Recently, other risk scores, such as the Freiburg Index for post‐TIPS survival (FIPS), have been developed and showed promising results [7, 8]. However, these scores only incorporate laboratory values as well as age, reflecting mostly liver and kidney function. Several aspects of the patient's clinical status and cirrhosis‐associated complications are not covered.

In the last decade, the importance of frailty in patients with cirrhosis has been extensively postulated [9, 10, 11]. Frailty is defined as a syndrome of decreased resistance to stressors and a multifactorial construct of a cumulative decline of physiologic reserve [12]. In patients with cirrhosis, frailty is common and associated with poorer prognosis and more frequent need for hospitalisations independent of the underlying liver function [13, 14].

Under consideration of the existing literature, frailty may be an appealing additional tool besides liver function for identifying patients with a poorer prognosis after TIPS insertion. However, the development and worsening of frailty in cirrhosis is related to ascites, anaemia (recurrent portal‐hypertensive bleeding) and sarcopenia, which are all closely linked to portal hypertension and subsequent inflammation [15]. TIPS leads to ascites control, reduces the risk for portal hypertensive bleeding and has been shown to improve sarcopenia [16]. Thus, a positive impact on frailty seems well possible.

So far, frailty has only been studied in the context of TIPS in one very small study including 12 patients and one study based on disease codes [17]. Therefore, this study aimed to investigate the impact of frailty on prognosis and the longitudinal changes of frailty over time in patients after elective TIPS insertion.

## 2 Patient and Methods

### 2.1 Study Population

In total, 123 patients with cirrhosis and available results in the Liver Frailty Index (LFI) were prospectively recruited in two study registers at Hannover Medical School (01/21–07/23) and the University Medical Center Mainz (03/22–01/24). This study represents a retrospective analysis of these prospectively recruited patients. Overall, 199 consecutive patients underwent TIPS insertion during the inclusion period. A study flowchart detailing the number of excluded patients and reason for exclusion can be found in Figure S1. As described elsewhere, patients with insufficient evidence of liver cirrhosis, with previous organ transplantation, severe neurological comorbidities or chronic renal impairment requiring haemodialysis were not considered for this study [18]. Additionally, only patients with the following TIPS indications were included: (i) refractory or recurrent ascites, (ii) hydrothorax, and (iii) failure of secondary prophylaxis with endoscopic variceal ligation plus non‐selective beta‐blockers after variceal bleeding according to the Baveno VII guidelines [19]. No patients needing an early or rescue TIPS due to variceal bleeding were approached for this study due to the different trajectory of the disease in these patients.

According to the predefined protocol, patients' data were collected from the respective medical records and by questioning them personally during their hospital stay before TIPS insertion. Follow‐up visits were conducted at 1, 3 and 6 months after TIPS insertion in the respective outpatient departments. The leading aetiology of the underlying liver disease was determined according to clinical, serological and histological findings. Diagnosis of cirrhosis was established by histology or a combination of conclusive appearance in ultrasound, radiological imaging, endoscopic features of portal hypertension and medical history. Blood biochemistry was assessed in all patients.

### 2.2 TIPS Insertion

The respective Departments of Diagnostic and Interventional Radiology at the Hannover Medical School and the University Medical Center Mainz performed TIPS insertion according to institutional standard operating procedures in all patients. The procedure was conducted under general anaesthesia using only polytetrafluoroethylene‐covered stent grafts (GORE VIATORR TIPS Endoprothesis, Flagstaff, Arizona, AZ, USA). Stent diameters were as follows: 6 mm (*n* = 34), 8 mm (*n* = 60) and 10 mm (*n* = 29).

### 2.3 Liver Frailty Index

Each patient was tested with the LFI prior to TIPS insertion to quantify physical functioning. The LFI consists of the following three physical tests and was instructed by trained study personnel:

Hand grip strength: the average of three trials, measured on the patient's dominant hand using a hand dynamometer.

Chair stands: measured as the number of seconds the patient needs to perform five chair stands with arms folded across the chest.

Balance testing: measured as the number of seconds the patient manages to balance in three positions (feet placed side‐to‐side, semi tandem and tandem) for a maximum time of 10 s each.

LFI was calculated based on the results of the administered tests, applying the online available calculator at http://liverfrailtyindex.ucsf.edu. Higher LFI values indicate a higher degree of physical functional impairment. Patients with a LFI value of ≥ 4.5 were considered frail, while a LFI between 3.2 and < 4.5 was considered pre‐frail and < 3.2 robust [20].

### 2.4 Diagnosis of HE

Testing for MHE was done using the portosystemic encephalopathy (PSE) syndrome test, which yields the psychometric hepatic encephalopathy score (PHES). Interpretation of PHES was done as previously described with German norms (version 2.0; 2020) [21, 22]. A score < −4 was considered pathological.

In addition, patients were tested with the Animal Naming Test (ANT) [18, 23, 24]. For the ANT, patients were asked to name as many animals as possible in 1 min. Repeats and errors are excluded from the calculations. The number of named animals after 1 min is defined as the score. To compensate for the influence of age and education, we calculated the S‐ANT1 which has been proposed by Campagna et al. [23].

### 2.5 Study Design and Endpoints

The primary endpoints of this study were death or liver transplantation as well as the development of overt hepatic encephalopathy (overt HE, OHE) after TIPS insertion. At each visit or during unplanned hospitalisations, each patient was examined by an experienced hepatologist to rule in or rule out OHE. Additionally, every patient was asked about hospitalisations in other hospitals. The presence of OHE was diagnosed after a detailed neurological examination according to the West‐Haven criteria.

As a secondary endpoint, patients were examined at predefined visits after 1, 3 and 6 months after TIPS insertion during outpatient appointments for the evaluation of longitudinal changes in LFI after TIPS.

### 2.6 Ethics

The studies were approved by the local ethics committees of Hannover Medical School (Nr. 8498_BO_S_2019) and the Landesärztekammer Rheinland‐Pfalz (Nr. 2021‐16247_1). Written informed consent was obtained from all participants. The study was carried out according to the principles of the Declaration of Helsinki.

### 2.7 Statistical Analysis

Data analysis was done with R 4.3.3 (R Core Team [2024]. R: A Language and Environment for Statistical Computing. R Foundation for Statistical Computing, Vienna, Austria. https://www.R‐project.org/) and RStudio version 2023.12.1.402 (Posit team [2024]. RStudio: Integrated Development Environment for R. Posit Software, PBC, Boston, MA. URL http://www.posit.co/). Categorical data are reported as numbers with percentages, continuous data as median with interquartile range (IQR). Pairwise comparisons were performed with the Wilcoxon rank sum test, Pearson's Chi‐squared test or the Fisher's exact test, as appropriate. Comparisons between paired groups were performed using Friedman's test. Spearman's rank correlation coefficient was used for correlation analyses. The reverse Kaplan–Meier method was used to calculate median follow‐up time.

The {tidycmprsk} R package (v0.2.0, Daniel D. Sjoberg and Teng Fei 2022) was used for both cumulative incidence functions for competing risk analyses and Fine and Gray competing risk regression analyses. Here liver transplantation/death were considered as competing events.

For liver transplantation‐free survival analysis, Kaplan–Meier curves and Cox proportional hazards regression models were built. The log‐rank test was used to test for differences between groups in Kaplan–Meier curve analysis. In all Cox analyses, a two‐state model with death and liver transplantation as composite endpoints was used (0: alive, not transplanted; 1: dead or liver transplanted). To assess non‐linear effects of LFI on transplantation‐free survival, LFI was fitted using restricted cubic splines with 4 knots in a Cox model ({rms} R package, v6.8–0, Harrell Jr. FE, 2024). For further analyses, the LFI was then divided into quartiles.

In case of missing data, a complete‐case analysis was made. A 0.05 level was chosen to define statistically significant deviations from the respective null hypothesis.

## 3 Results

In total, 123 patients with cirrhosis and TIPS were included in this study. The cohort was predominantly male (68.3%) with a median age of 59 years (IQR 53; 65). The most frequent indication for TIPS was ascites (69.9%). At study inclusion prior to TIPS insertion, median LFI was 4.32 (IQR 3.78; 4.88) and 40.7% of the patients were considered frail, while 51.2% were pre‐frail and only 8.1% robust. Demographics and clinical characteristics of the cohort are displayed in Table 1. Most patients had a modified TIPS score (MOTS) of 0 points (Table 1) [25].

**TABLE 1:** Demographics and baseline characteristics of the study cohort.

Variable	*N*	Total cohort, *N* = 123
Age (years)	123	59 (53, 65)
Gender		
Male	123	84 (68%)
Female		39 (32%)
Aetiology		
ALD	123	62 (50%)
MetALD		7 (5.7%)
MASLD		16 (13%)
Viral		4 (3.3%)
Other		18 (15%)
Mixed		16 (13%)
Indication for TIPS		
Ascites	123	86 (70%)
Bleeding		23 (19%)
Hydrothorax		1 (0.8%)
Ascites + bleeding		10 (8.1%)
Ascites + hydrothorax		2 (1.6%)
Other		1 (0.8%)
Liver Frailty Index (LFI)	123	4.32 (3.78, 4.88)
LFI categorised		
Robust	123	10 (8.1%)
Pre‐frail		63 (51%)
Frail		50 (41%)
CHE (PHES)		
CHE−	118	61 (52%)
CHE+		57 (48%)
PHES	118	−4.0 (−7.0, −2.0)
S‐ANT1	120	19 (15, 24)
Child–Pugh		
A	123	11 (8.9%)
B		102 (83%)
C		10 (8.1%)
FIPS score	120	−0.07 (−0.79, 0.39)
MELD score	123	12.0 (9.0, 16.0)
History of OHE	123	32 (26%)
Sodium (mmol/L)	121	136.0 (133.0, 138.0)
Creatinine (mg/dL)	123	1.20 (0.88, 1.68)
Bilirubin (mg/dL)	123	1.00 (0.62, 1.50)
Cholinesterase (kU/L)	115	2.94 (2.33, 4.84)
AST (U/L)	121	36 (29, 50)
ALT (U/L)	120	20 (14, 31)
Albumin (g/L)	120	31.0 (27.0, 35.3)
CRP (mg/L)	120	8 (4, 20)
WBC (per nL)	120	5.10 (3.60, 7.83)
Haemoglobin (g/dL)	120	10.05 (8.40, 12.03)
Platelets (per nL)	120	117 (75, 180)
INR	123	1.21 (1.13, 1.33)
Lactulose		
No	123	36 (29%)
Yes		87 (71%)
Rifaximin		
No	123	56 (46%)
Yes		67 (54%)
Modified TIPS‐Score		
0	51	43 (84%)
1		6 (12%)
2		2 (3.9%)

Additionally, a comparison between frail, pre‐frail and robust patients is displayed in Table S1, and a comparison between patients from Mainz and Hannover is displayed in Table S2.

At baseline, there was a weak positive correlation between performance in LFI and age or CRP, while there was a weak negative correlation between LFI and albumin or performance in PHES or S‐ANT1 (Figure S2).

### 3.1 LFI and Death/Liver Transplantation After TIPS Insertion

During follow‐up, 24 (19.5%) patients died and 6 (4.9%) underwent liver transplantation. In the subsequent analyses, we investigated the impact of frailty/LFI on the occurrence of the composite endpoint of death and need for liver transplantation as well as survival under consideration of liver transplantation as a competing event.

**FIGURE 1:**
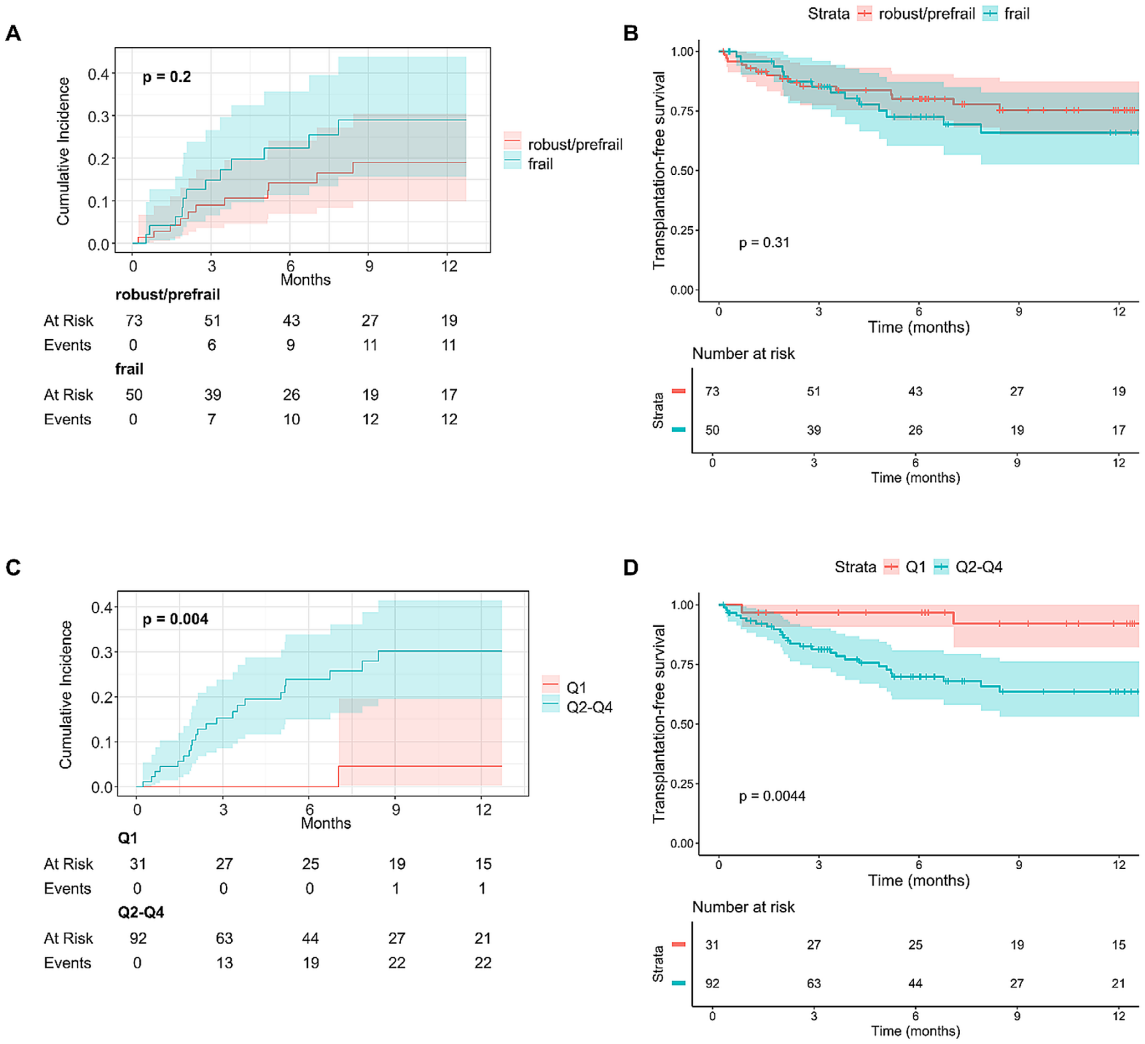
Cumulative incidence of death under consideration of liver transplantation as competing event (left, A/C) and liver transplantation‐free survival (right, B/D) in patients stratified by the Liver Frailty Index in (A/B) frail vs. non‐frail and (C/D) in Quartile 1 (Q1) vs Quartile 2–4 (Q2–Q4).

In univariable analyses, overall survival and transplantation‐free survival did not differ between frail and non‐frail patients (Figure 1A/B). Of note, when stratifying the cohort into robust, pre‐frail and frail patients, there was a numerical difference regarding the prognosis between robust and pre‐frail or frail patients (Figure S3). Remarkably, no robust patient (*n* = 10) died or received a liver transplantation during follow‐up. We also stratified the cohort according to quartiles in LFI and compared the prognosis between patients within Q1 to those within Q2–Q4. Here, prognosis was significantly better in patients with an LFI in Q1 compared to the other quartiles (Figure 1C/D).

To gain further insights into gender differences, we repeated these analyses in men and women, respectively. Of note, female patients had significantly higher LFI values compared to male patients (median LFI 4.55 [IQR 4.01, 5.34] vs. 4.25 [IQR 3.69, 4.62], *p* = 0.027). We found no differences in transplantation‐free survival between robust/prefrail and frail patients in either sex (Figure S4). Male patients with an LFI in Q1 had a significantly longer transplantation‐free survival compared to male patients in Q2–Q4, while there was no difference in female patients (Figure S5). However, these results should be interpreted with caution due to the small number of female patients (*n* = 39) and consecutive small number of outcome events.

Next, we analysed non‐linear effects of LFI on transplantation‐free survival in a univariable Cox model using restricted cubic splines with four knots (Figure S6). Here, LFI showed a lower relative hazard with a linear relation until 3.5–4, with a consecutive flattening of the curve.

Univariable Fine and Gray‐ and Cox‐regression analyses for the outcomes of overall survival or the composite endpoint are displayed in Tables S3 and S4. Here, LFI as a metric variable was not associated with a poorer prognosis, while there was a strong association between LFI in Q1 and a better prognosis in multivariable analyses (Table 2). In multivariable Cox regression analysis, no significant association between frailty and Transplantation‐free survival was found in the subgroup of patients with ascites as TIPS indication (Table S5).

**TABLE 2:** Multivariable Fine and Gray regression (top) for survival analyses and Cox regression (bottom) for liver transplantation‐free survival analyses.

Variable	*N*	sHR	95% CI	*p*
LFI (cont.)	120	1.39	0.97, 2.00	0.075
FIPS score	120	2.00	1.26, 3.17	0.003
LFI (cat.)				
Q1	30	—	—	
Q2–Q4	90	8.57	1.20, 61.2	0.032
FIPS score	120	1.97	1.23, 3.15	0.005

Variable	*N*	HR	95% CI	*p*
LFI (cont.)	120	1.21	0.82, 1.80	0.3
FIPS score	120	2.50	1.50, 4.15	< 0.001
LFI (cat.)				
Q1	30	—	—	
Q2–Q4	90	5.00	1.18, 21.2	0.029
FIPS score	120	2.45	1.47, 4.06	< 0.001

*Note*: LFI categorised in robust, pre‐frail and frail not shown because no patient died in the robust group. In multivariable Fine and Gray regression for survival analyses, liver transplantation was treated as a competing event.

Abbreviations: cat., categorical; CI, confidence interval; cont., continuous; FIPS, Freiburg index of post‐TIPS survival; LFI, Liver Frailty Index; Q, quartile; sHR, subdistribution hazard ratio.

### 3.2 LFI and Development of OHE After TIPS Insertion

All 123 patients were followed for the development of the first OHE episode after enrollment or death/liver transplantation. Median follow‐up time was 317 days (95% CI: 209; 370). In total, 40 (32.5%) patients developed at least one OHE episode during follow‐up. The distribution of OHE grades was as follows: 26 (65%) HE grade II, 13 (32.5%) HE grade III, 1 (2.5%) HE grade IV. The majority of OHE events (37/40, 92.5%) occurred during the first 3 months after TIPS insertion (Figure S7). Additionally, 17 (13.8%) patients died (*n* = 14) or received a liver transplantation (*n* = 3) before an episode of OHE.

**FIGURE 2:**
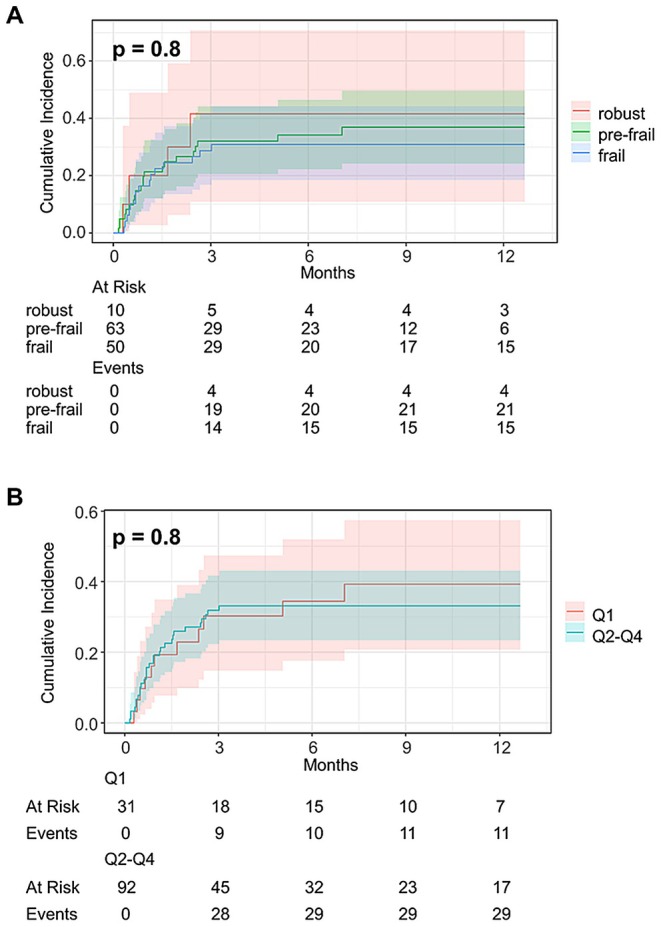
Cumulative overt HE (OHE) incidence. Cumulative OHE incidence in (A) patients stratified by the Liver Frailty Index (robust vs. pre‐frail vs. frail, *p* = 0.8), (B) patients stratified by the Liver Frailty Index (Quartile 1 [Q1] vs. Quartile 2–4 [Q2–Q4], *p* = 0.8).

In the total cohort, the cumulative OHE incidences did not differ between robust, pre‐frail or frail patients (*p* = 0.8, Figure 2A). The same was noted when comparing patients with LFI results in the first quartile (Q1, LFI ≤ 3.77) compared to patients with LFI results in the other three quartiles (Q2‐4; Q2: LFI > 3.77 and ≤ 4.32; Q3: LFI > 4.32 and ≤ 4.88; Q4: LFI > 4.88) (*p* = 0.8, Figure 2B). In addition, cumulative OHE incidence stratified by frailty status (robust/prefrail vs. frail and Q1 vs. Q2–Q4) showed no differences after splitting the cohort into female and male patients (not shown).

To identify variables independently associated with the development of OHE, we fitted multivariable regression models using the method of Fine and Gray. Due to the limited number of outcome events (OHE) and to avoid overfitting, we decided to build the multivariable models based on predefined known risk factors for OHE development (FIPS and decrease of portosystemic gradient (PSG) in percent) and LFI. In the total cohort, performance in LFI was not associated with a higher risk of OHE in multivariable regression analysis (sHR 0.80, 95% CI 0.57; 1.14, *p* = 0.2, Table 3) after adjusting for decrease of PSG and FIPS. This finding also remained robust when dichotomising LFI into the group within the lowest quartile and the group in the three other quartiles in a separate regression model (*p* = 0.7) (Table 3). In addition, no association was found between frailty and OHE risk in the subgroup of patients with ascites as TIPS indication (Table S6). Results of univariable Fine and Gray regression analyses are provided in Table S7. Here, we also analysed results for the subtests of LFI, which were not significantly associated with the occurrence of post‐TIPS OHE (Table S8).

**TABLE 3:** Multivariable Fine and Gray regression analysis for OHE development.

Variable	*N*	sHR	95% CI	*p*
LFI (cont.)	120	0.80	0.57, 1.14	0.2
PSG delta (%)	120	7.17	1.54, 33.4	0.012
FIPS score	120	1.79	1.21, 2.66	0.004
LFI (cat.)				
Robust	10	—	—	
Pre‐frail	60	0.57	0.17, 1.93	0.4
Frail	50	0.46	0.13, 1.66	0.2
PSG delta (%)	120	6.63	1.33, 32.9	0.021
FIPS score	120	1.80	1.21, 2.68	0.004
LFI (cat.)				
Q1	30	—	—	
Q2–Q4	90	0.85	0.42, 1.71	0.7
PSG delta (%)	120	6.81	1.39, 33.3	0.018
FIPS score	120	1.69	1.17, 2.44	0.005

Abbreviations: cat., categorical; CI, confidence interval; cont., continuous; FIPS, Freiburg index of post‐TIPS survival; LFI, Liver Frailty Index; PSG, portosystemic gradient; Q, quartile; sHR, subdistribution hazard ratio.

### 3.3 Changes in LFI After TIPS Insertion

**FIGURE 3:**
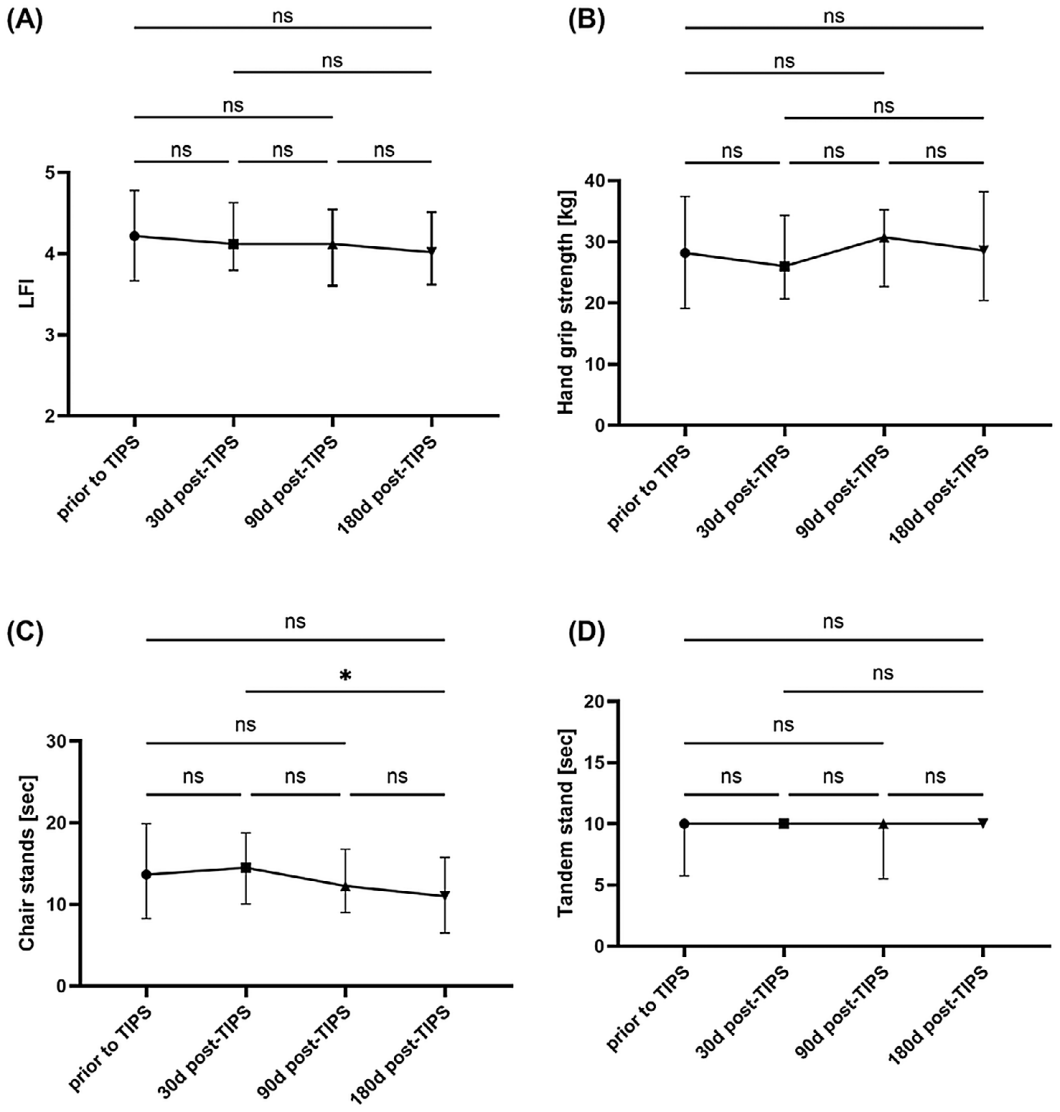
Longitudinal changes in Liver Frailty Index (LFI) and its subtests after TIPS insertion. (A) The trajectory of LFI after TIPS insertion, (B) the trajectory of hand grip strength, (C) the trajectory of time for chair stands and (D) the time of the tandem stand. Only patients with LFI results available at all four time points have been included (*n* = 41). *p < 0.05.

In 41 patients, LFI results were available at baseline and all other follow‐up time points 1, 3 and 6 months after TIPS insertion. Baseline characteristics of these patients are displayed in Table S9. In the total cohort, there was no significant change in LFI when comparing the various time points (Figure 3). The same was found when analysing changes in the subtests of LFI (hand grip strength, chair stands or balance testing [tandem stand]) (Figure 3).

**FIGURE 4:**
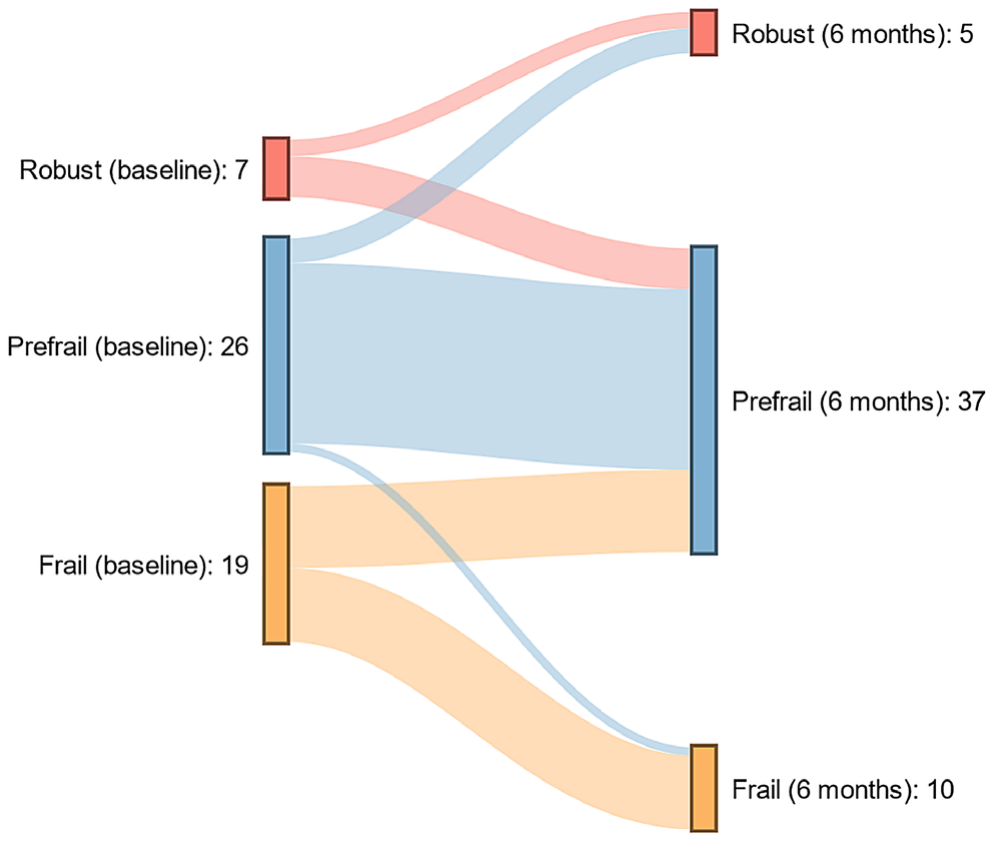
Sankey plot displaying the trajectory of frailty in patients with available data 6 months after TIPS insertion. All patients with LFI measurements before TIPS insertion and after 6 months of follow‐up were included in the analysis (*n* = 52).

In 52 patients, LFI results were available at baseline and 6 months after TIPS insertion. Baseline characteristics of these patients are displayed in Table S10. In total, 13 patients (24.5%) improved in LFI 6 months after TIPS insertion, while six patients (11.5%) deteriorated in LFI. The trajectory of frail, pre‐frail or robust patients within 6 months after TIPS insertion is displayed in Figure 4. To identify baseline variables associated with an improvement in LFI 6 months after TIPS insertion, we built a multivariable linear regression model using a stepwise variable selection procedure including baseline variables. Here, a higher LFI (*β* = −0.667, *p* < 0.001) and younger age (*β* = 0.364, *p* = 0.005) at baseline were associated with an improvement in LFI 6 months after TIPS insertion (Table 4).

**TABLE 4:** Multivariable linear regression analysis for the association between changes in LFI within 6 months after TIPS insertion and characteristics prior to TIPS.

Characteristics	*N*	*β*	Regression coefficient	*p*
LFI	52	−0.67	−0.57	< 0.001
Age	52	0.36	0.03	0.005

*Note*: Outcome: delta LFI (LFI 6 months after TIPS–LFI prior to TIPS), a negative delta LFI indicates an improvement in LFI, a positive delta LFI indicates a worsening in LFI after TIPS. *R* ^2^ = 0.42. The model was built based on a stepwise variable selection procedure. Not significant were as follows: PHES (*p* = 0.05), gender (*p* = 0.73), PSG delta (*p* = 0.17), FIPS (*p* = 0.76), history of OHE (*p* = 0.83), sodium (*p* = 0.16), albumin (*p* = 0.10), platelets (*p* = 0.36) and haemoglobin (*p* = 0.84).

## 4 Discussion

In this bi‐centric study, we provide detailed insight into the longitudinal evolution of frailty after TIPS insertion in patients with cirrhosis as well as the prognostic utility of performance in LFI prior to TIPS. We found no significant improvement in LFI in longitudinal analyses in the total cohort after TIPS insertion. However, there was a significant improvement in LFI in patients with poorer physical functioning prior to TIPS insertion. Moreover, we found that patients within the first quartile of LFI prior to TIPS had an excellent and significantly better prognosis than patients in the other three quartiles. In contrast, there was no association between performance in LFI and the risk for post‐TIPS OHE.

Frailty has been linked to several complications of cirrhosis in the past and may be especially linked to a higher risk of HE and mortality [9, 26, 27]. In patients with decompensated cirrhosis, frailty is a common phenomenon, but its prevalence depends heavily on the test used to define frailty [15]. In our current study, 50 (41%) patients were classified as frail according to the LFI and only 10 (8%) were robust. When setting these numbers into context, then the prevalence of frailty in our cohort is comparatively high. A large multicentre US‐based study by Lai et al. investigated the LFI in 1044 patients on the liver transplantation waitlist and found a prevalence of frailty according to LFI of 25% [9]. In the pivotal study developing the LFI, the cut‐off defining frailty was set at the 80th percentile of a cohort comprising 536 patients with cirrhosis [20]. Additionally, the higher prevalence of ascites in our cohort (80% vs. 34% in the original study by Lai et al.) may partially explain the higher observed frailty prevalence [20]. Taken together, when interpreting our findings, one has to keep in mind that our bi‐centric TIPS cohort already suffered from a comparably poor physical functioning prior to TIPS insertion.

Muscle quality and quantity play an important role in the natural course of liver cirrhosis and sarcopenia in the context of TIPS insertion and have been extensively studied. Several studies demonstrated that sarcopenia is associated with a poorer prognosis and higher risk of OHE after TIPS insertion [28, 29]. Pathophysiologically, this makes sense given that muscle plays an important role in ammonia metabolism and patients with sarcopenia are more susceptible to complications of cirrhosis including infections [30]. However, various studies also found that sarcopenia is reversible after TIPS insertion and improvements in muscle quantity have been linked to lower ammonia levels as well as a better performance in PHES [31]. That resulted in the hypothesis that TIPS insertion might also reverse frailty in patients with cirrhosis. To our own surprise, we did not detect a significant improvement in LFI in the total cohort at none of the follow‐up time points in patients with at least 6 months of follow‐up. This is in line with a small study investigating the evolution of frailty in 12 patients with TIPS insertion from a single centre in Australia [17]. The large sample size of our study enabled subgroup analyses and the identification of predictors for changes in LFI after TIPS insertion. Here, the strongest predictor for changes in LFI after TIPS was LFI at baseline, which means that the physical function of patients with high LFI at baseline could be improved by TIPS insertion. This indicates that elements relevant to reduced physical functioning can at least partly be addressed by reversal of portal hypertension in these patients. In this context, one hypothesis is the reduction of systemic inflammation through effective treatment of portal hypertension, which in turn could be relevant for improving physical functioning.

To the best of our knowledge, the impact of frailty on clinical outcomes after TIPS insertion has not been studied in detail. Our study demonstrates that the established cut‐offs for frailty or LFI as a metric variable are neither associated with a higher risk for OHE after TIPS nor with poorer liver transplantation‐free survival. At first sight, this finding is in contrast with several studies linking sarcopenia to poorer outcomes after TIPS [29, 32]. However, our study underlines the fact that sarcopenia is not equivalent to frailty in patients with cirrhosis, given that the concept of frailty is not only based on muscle quantity but also on muscle functioning as well as cognitive function. Of note, when analysing our data, it has to be acknowledged that there might be a relevant difference in prognosis between patients classified as robust and those classified as pre‐frail/frail. No patient classified as robust prior to TIPS died or needed a liver transplantation during follow‐up. Additionally, we found a significantly better prognosis regarding liver transplantation‐free survival for patients in the lowest LFI quartile prior to TIPS insertion compared to patients in the other three quartiles. Looking at the restricted cubic spline for LFI, a clear ceiling effect can be seen at the boundary between the first and second quartiles of the cohort. This indicates that the established cut‐offs for LFI classifying patients with cirrhosis as robust, pre‐frail or frail might not be applicable for risk stratifying patients with elective TIPS insertion [20].

The findings of our study have important, direct implications for managing patients with cirrhosis and eligibility for elective TIPS insertion. First and foremost, our results suggest that TIPS insertion should not be withheld in patients with frailty, because prognosis is comparable to patients with pre‐frailty according to the established LFI cut‐offs and TIPS might even improve physical functioning in these patients in the long term. Second, implementation of LFI prior to TIPS into the routine evaluation process enables identifying patients with an excellent prognosis and adds a more granular risk stratification independent of other prognostic tools, such as MELD or FIPS [7].

Our study has limitations. First, we have to acknowledge that a follow‐up period of 6 months may be too short to identify improvements in LFI, especially in patients with better LFI at baseline. Changes in this subgroup might only become detectable after long‐term follow‐up. Second, not all patients with available baseline data had LFI data available 6 months after TIPS insertion. This is explained by the characteristics of this special cohort: some patients died or received liver transplantation, and some were lost to follow‐up. This leads to a selection bias towards patients being able to attend the follow‐up visit and thus to the exclusion of patients who died before or were incompliant and did not attend the visit. This has to be taken into account, especially for the group of frail patients who have a higher risk for complications. However, not all patients who were classified as robust or prefrail had LFI data available 6 months after TIPS insertion. Therefore, this bias is not exclusive to frail patients. Third, although most patients underwent paracentesis before LFI testing, residual ascites prior to TIPS insertion may have affected patients' performance in the LFI (e.g., chair stands) due to its physical barrier, and post‐TIPS improvement may be partly due to the removal of this physical barrier.

In conclusion, we provide detailed insight in the longitudinal evolution of frailty after TIPS insertion in patients with cirrhosis and were able to demonstrate that improvement in LFI can be achieved in patients with poor physical functioning prior to TIPS insertion. Additionally, we found that patients within the first quartile of LFI prior to TIPS had an excellent and significantly better prognosis than patients in the other three quartiles. While poor LFI should not be considered a contraindication for TIPS, it might allow a more granular risk stratification independent of other established prognostic tools, such as MELD or FIPS.

## Author Contributions

**Martin Andreas Kabelitz:** investigation, conceptualization, formal analysis, writing – original draft, writing – review and editing, methodology, data curation, software, validation. **Simon Johannes Gairing:** conceptualization, methodology, formal analysis, writing – original draft, writing – review and editing, investigation, visualization, data curation, software, validation. **Anja Tiede:** writing – review and editing, data curation. **Eva Maria Schleicher:** writing – review and editing. **Liv Grete Ahl:** writing – review and editing, data curation. **Lea Wagner:** writing – review and editing, data curation. **Falko Zucker‐Reimann:** writing – review and editing, data curation. **Hannah Rieland:** writing – review and editing, data curation. **Jim Benjamin Mauz:** writing – review and editing, data curation. **Julia Weinmann‐Menke:** writing – review and editing, data curation. **Bernhard C. Meyer:** writing – review and editing. **Michael Bernhard Pitton:** writing – review and editing. **Heiner Wedemeyer:** writing – review and editing, funding acquisition, resources. **Peter Robert Galle:** writing – review and editing, funding acquisition, resources. **Lisa Sandmann:** writing – review and editing, writing – original draft, investigation, conceptualization, resources. **Benjamin Maasoumy:** writing – original draft, writing – review and editing, investigation, conceptualization, funding acquisition, methodology, project administration, supervision, resources. **Christian Labenz:** writing – original draft, writing – review and editing, investigation, conceptualization, funding acquisition, methodology, project administration, supervision, resources.

## Conflicts of Interest

S.J.G.: Travel expenses: Ipsen and Gilead. H.W.: Lecture fees and consulting: Abbott, Bristol‐Myers‐Squibb, Hoffmann‐La Roche, Roche, Gilead, GlaxoSmithKline, Janssen, Vir Biotechnology. Research support: Abbott and Biotest. P.R.G.: Lecture fees and consulting: Merz Pharmaceuticals. L.S.: Lecture and consultant fees: Falk Pharma e.V., Roche and Gilead. Travel expenses: Abbvie. B.M.: Lecture and consultant fees: AbbVie, Fujirebio, Gilead, Luvos, MSD, Norgine, Roche, W. L. Gore & Associates. Research grants: Altona, EWIMED, Fujirebio and Roche. C.L.: Lecture and consultant fees: Merz Therapeutics, Norgine, Intercept, Gilead Sciences, Falk Foundation e.V., CSL Behring, Boehringer Ingelheim. Research grants: Merz Therapeutics, Norgine. The other authors declare no conflicts of interest.

## Supporting information


**Data S1:** apt70315‐sup‐0001‐supinfo.docx.

## Data Availability

The data that support the findings of this study are available on request from the corresponding author. The data are not publicly available due to privacy or ethical restrictions.
