# Supplementary material for: Impact of Frailty on the Prognosis of Patients With Liver Cirrhosis Undergoing Insertion of a TIPS
Source: Aliment Pharmacol Ther. 2025 Aug 1;63(1):109–18. doi: 10.1111/apt.70315 (PMC12690237; doi:10.1111/apt.70315)
Supplement: Supplementary file 1 — Data S1: apt70315‐sup‐0001‐supinfo.docx. [file APT-63-109-s001.docx]

**Impact of frailty on the prognosis of patients with liver cirrhosis undergoing insertion of a transjugular intrahepatic portosystemic shunt**

Martin Andreas Kabelitz^1^*, Simon Johannes Gairing^2,3*^, Anja Tiede^1,4^, Eva Maria Schleicher^2,3^, Liv Grete Ahl^1^, Lea Wagner^1^, Falko Zucker-Reimann^2^, Hannah Schneider^1^, Jim Mauz^1^, Julia Weinmann-Menke^2,3^, Michael Bernhard Pitton^2,3^, Heiner Wedemeyer^1,4^, Peter Robert Galle^2^, Lisa Sandmann^1†^, Benjamin Maasoumy^1,4†^, Christian Labenz^2,3†^

**Table of contents**

[Figures 3](#_Toc202282820)

[Fig. S1. Flow chart depicting patient selection 3](#_Toc202282821)

[Fig. S2. Correlation between Liver Frailty Index (LFI) and various clinical characteristics at baseline. 4](#_Toc202282822)

[Fig. S3. Cumulative incidence of death (left, A) and transplantation-free survival (right, B) in patients stratified by the Liver Frailty Index in robust vs prefrail vs frail. 5](#_Toc202282823)

[Fig. S4. Transplantation-free survival dichotomized by frailty status (robust/prefrail vs frail) in male (left) and female (right) patients. 6](#_Toc202282824)

[Fig. S5. Transplantation-free survival dichotomized by frailty status (Q1 vs Q2-Q4) in male (left) and female (right) patients. 6](#_Toc202282825)

[Fig. S6. Non-linear effect of LFI on transplantation-free survival. 7](#_Toc202282826)

[Fig. S7. Density plot of post-TIPS OHE events. 8](#_Toc202282827)

[Tables 9](#_Toc202282828)

[Table S1: Baseline characteristics of patients stratified by frailty according to the Liver Frailty Index (LFI) 9](#_Toc202282829)

[Table S2: Baseline characteristics of patients stratified by center 11](#_Toc202282830)

[Table S3. Univariable Fine and Gray competing risk regression analysis for the association of variables with death under consideration of liver transplantation as competing event. 13](#_Toc202282831)

[Table S4. Univariable Cox models for the association of variables with transplantation-free survival. 15](#_Toc202282832)

[Table S5. Multivariable Cox regression analysis for Transplantation-free survival in the subgroup of patients with ascites as indication for TIPS 17](#_Toc202282833)

[Table S6. Multivariable Fine and Gray competing risk regression analysis for the development of OHE in the subgroup of patients with ascites as indication for TIPS 18](#_Toc202282834)

[Table S7. Univariable Fine and Gray competing risk regression analysis for the development of OHE 19](#_Toc202282835)

[Table S8. Univariable Fine and Gray competing risk regression analysis for the development of OHE (subtests of the LFI) 21](#_Toc202282836)

[Table S9: Baseline characteristics of patients with available LFI data at 0, 1, 3, and 6 months (n = 41) 22](#_Toc202282837)

[Table S10: Baseline characteristics of patients with available LFI data at 0 and 6 months (n = 52) 24](#_Toc202282838)

# Figures

**
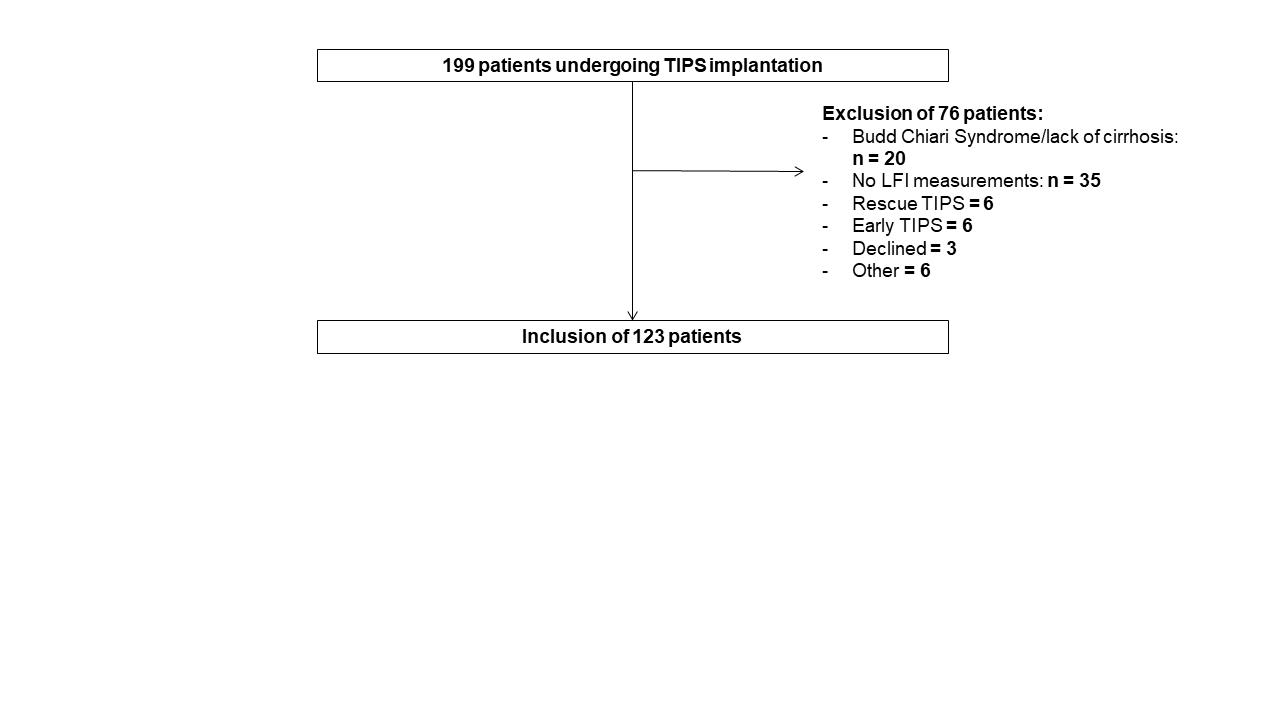
**

## Fig. S1. Flow chart depicting patient selection

Abbr.: LFI, Liver Frailty Index; TIPS, transjugular intrahepatic portosystemic shunt


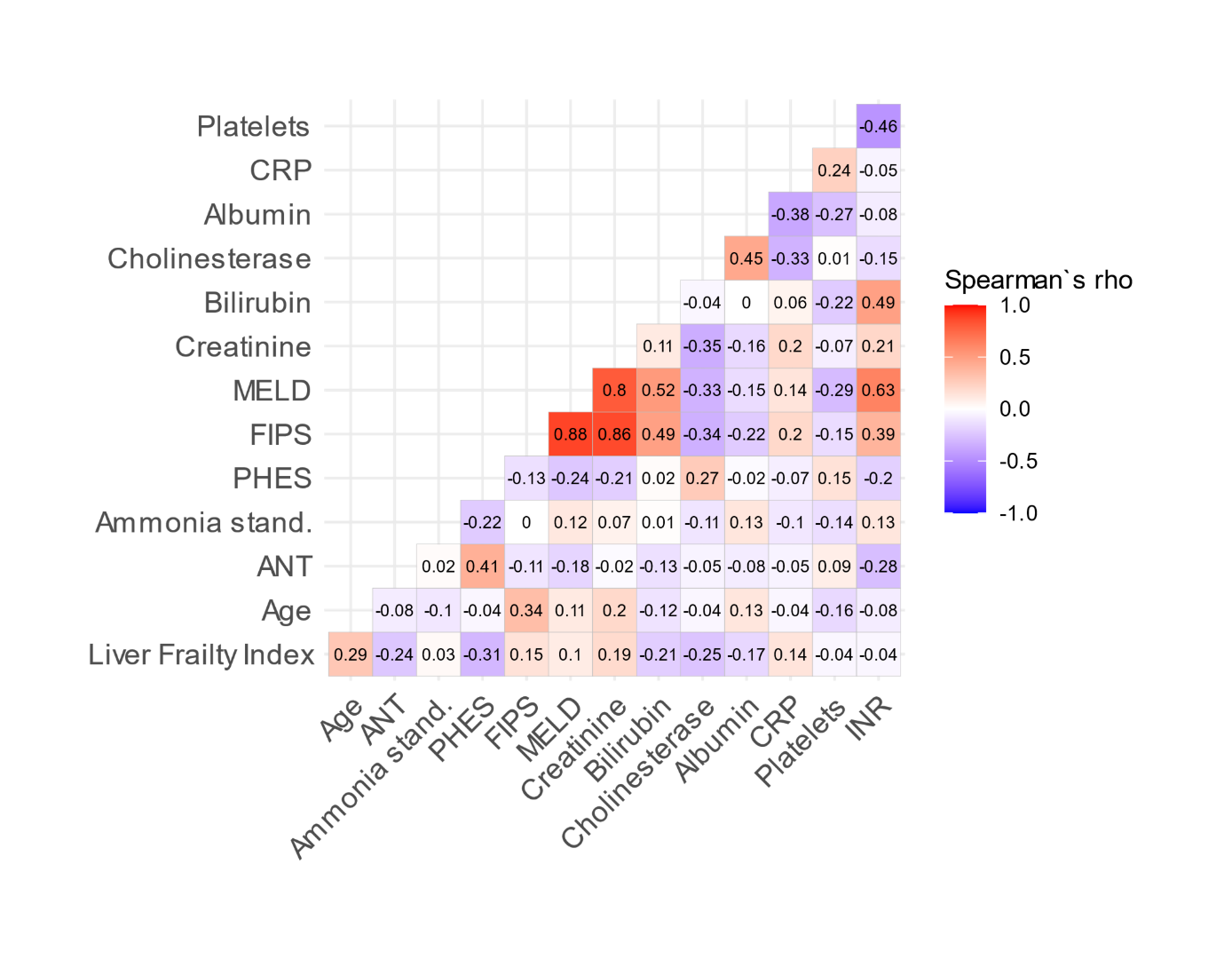


## Fig. S2. Correlation between Liver Frailty Index (LFI) and various clinical characteristics at baseline.

Abbr.: CRP, C-reactive protein; MELD, model for end-stage liver disease; FIPS, Freiburg index of post-TIPS survival; PHES, psychometric hepatic encephalopathy score; stand., standardized; ANT, Animal Naming Test; INR, International Normalized Ratio


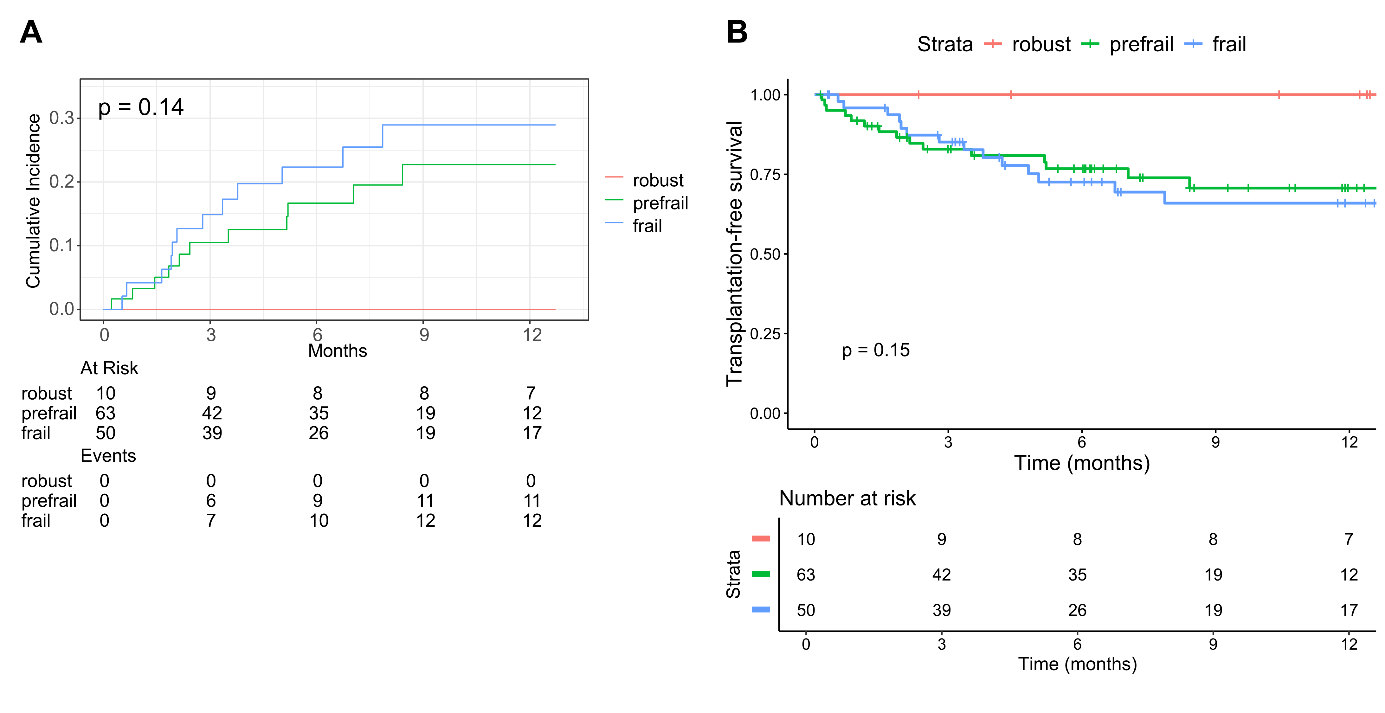


## Fig. S3. Cumulative incidence of death (left, A) and transplantation-free survival (right, B) in patients stratified by the Liver Frailty Index in robust vs prefrail vs frail.


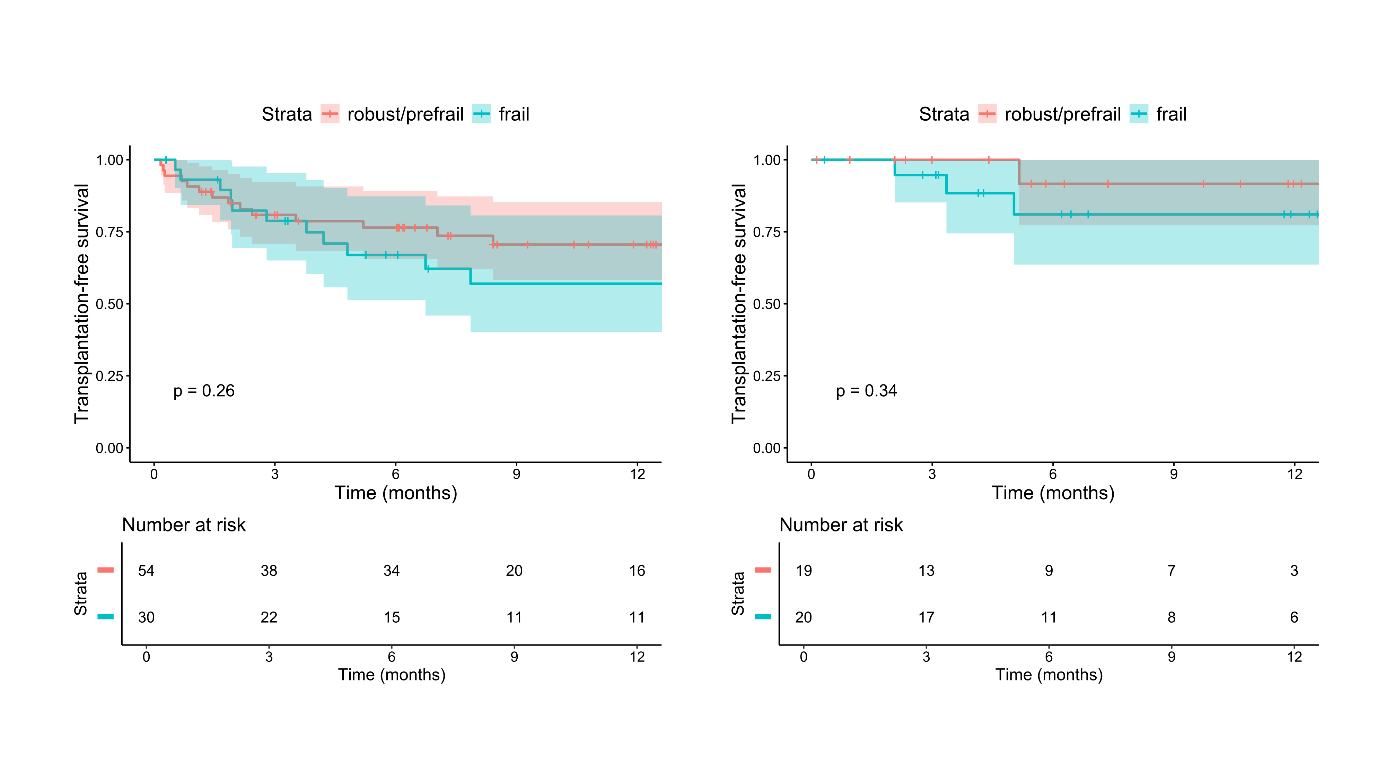


## Fig. S4. Transplantation-free survival dichotomized by frailty status (robust/prefrail vs frail) in male (left) and female (right) patients.


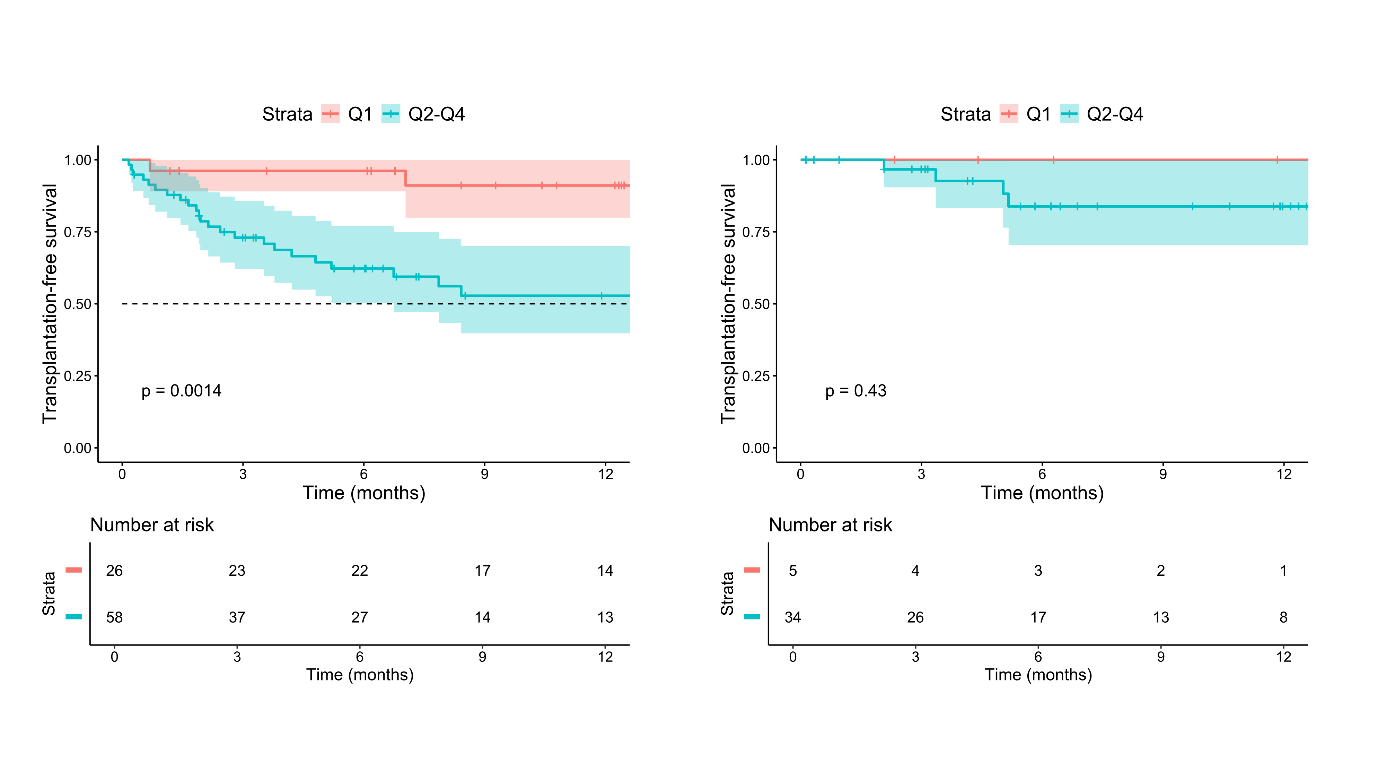


## Fig. S5. Transplantation-free survival dichotomized by frailty status (Q1 vs Q2-Q4) in male (left) and female (right) patients.


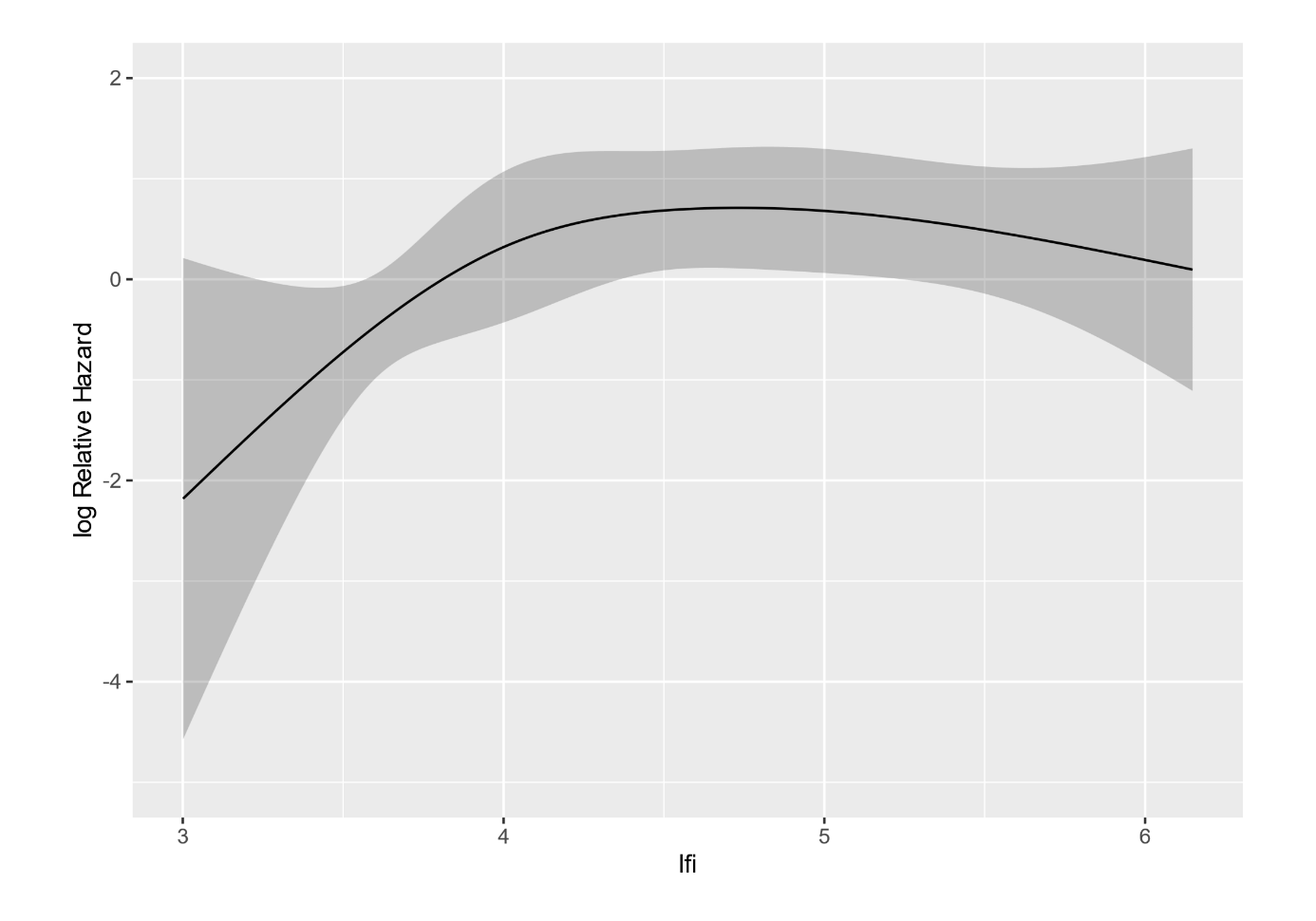


## Fig. S6. Non-linear effect of LFI on transplantation-free survival.

An univariable Cox model with LFI as restricted cubic spline with four knots was fitted.


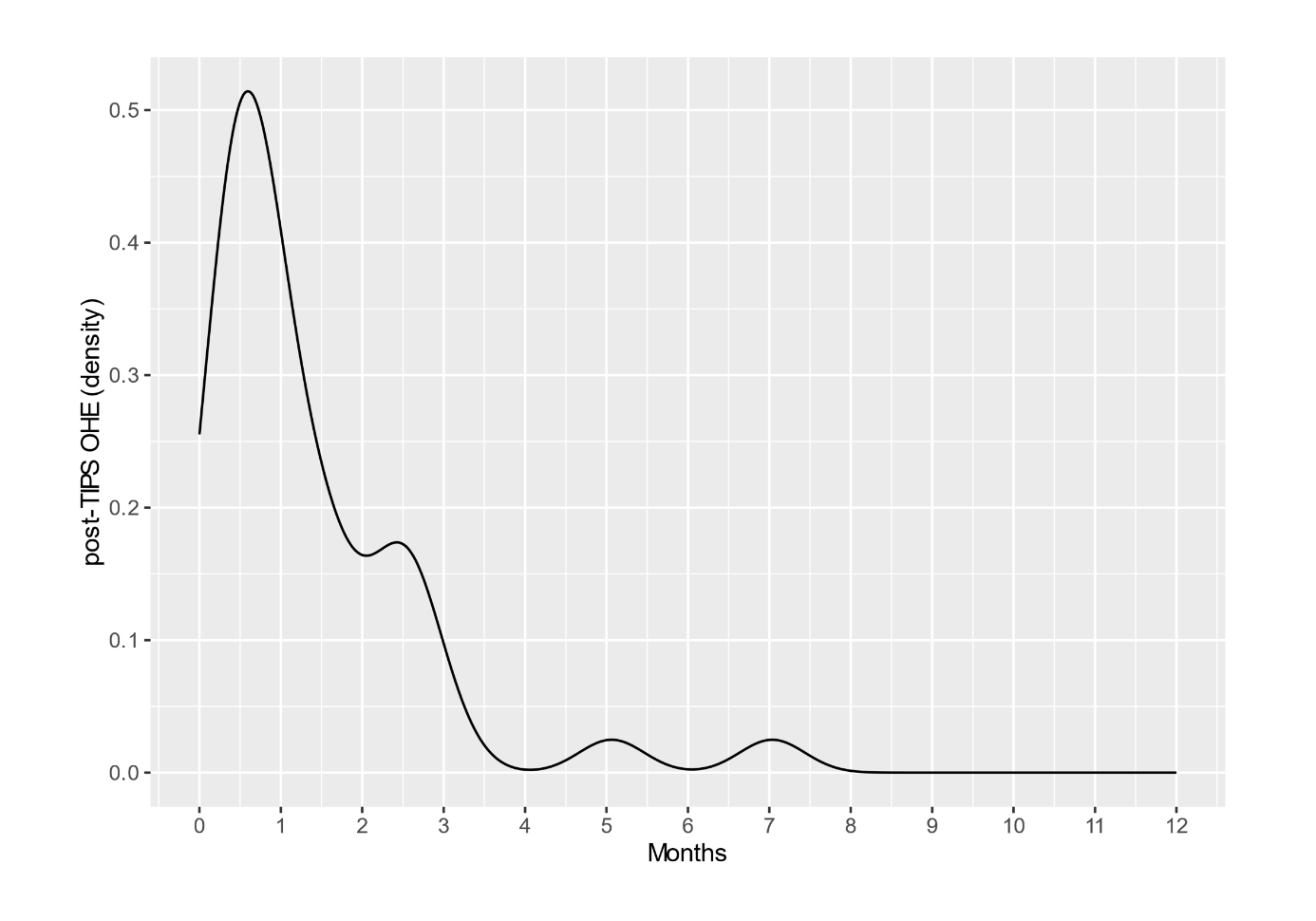


## Fig. S7. Density plot of post-TIPS OHE events.

# Tables

## Table S1: Baseline characteristics of patients stratified by frailty according to the Liver Frailty Index (LFI)

| **Variable** | **N** | **Robust**  N = 10 | **Pre-frail**  N = 63 | **Frail**  N = 50 | **p-value** |
| --- | --- | --- | --- | --- | --- |
| **Age (years)** | 123 | 50 (41, 63) | 60 (52, 67) | 60 (55, 65) | 0.2 |
| **Gender** | 123 |  |  |  | 0.2 |
| male |  | 8 (80%) | 46 (73%) | 30 (60%) |  |
| female |  | 2 (20%) | 17 (27%) | 20 (40%) |  |
| **Etiology** | 123 |  |  |  | 0.2 |
| ALD |  | 2 (20%) | 34 (54%) | 26 (52%) |  |
| MetALD |  | 0 (0%) | 4 (6.3%) | 3 (6.0%) |  |
| MASLD |  | 1 (10%) | 9 (14%) | 6 (12%) |  |
| Viral |  | 2 (20%) | 1 (1.6%) | 1 (2.0%) |  |
| Other |  | 2 (20%) | 7 (11%) | 9 (18%) |  |
| Mixed |  | 3 (30%) | 8 (13%) | 5 (10%) |  |
| **Indication for TIPS** | 123 |  |  |  | 0.060 |
| Ascites |  | 4 (40%) | 47 (75%) | 35 (70%) |  |
| Bleeding |  | 6 (60%) | 9 (14%) | 8 (16%) |  |
| Hydrothorax |  | 0 (0%) | 1 (1.6%) | 0 (0%) |  |
| Ascites + bleeding |  | 0 (0%) | 4 (6.3%) | 6 (12%) |  |
| Ascites + hydrothorax |  | 0 (0%) | 2 (3.2%) | 0 (0%) |  |
| Other |  | 0 (0%) | 0 (0%) | 1 (2.0%) |  |
| **Liver Frailty Index (LFI)** | 123 | 2.87 (2.52, 3.01) | 3.98 (3.70, 4.25) | 5.14 (4.71, 5.69) | **<0.001** |
| **CHE (PHES)** | 118 |  |  |  | **0.015** |
| CHE- |  | 8 (80%) | 36 (58%) | 17 (37%) |  |
| CHE+ |  | 2 (20%) | 26 (42%) | 29 (63%) |  |
| **PHES** | 118 | -3.0 (-4.0, -2.0) | -4.0 (-7.0, -1.0) | -5.0 (-9.0, -2.3) | **0.047** |
| **S-ANT1** | 120 | 23 (16, 30) | 19 (16, 26) | 18 (14, 22) | 0.2 |
| **Child Pugh** | 123 |  |  |  | **0.005** |
| A |  | 5 (50%) | 4 (6.3%) | 2 (4.0%) |  |
| B |  | 5 (50%) | 53 (84%) | 44 (88%) |  |
| C |  | 0 (0%) | 6 (9.5%) | 4 (8.0%) |  |
| **FIPS score** | 120 | -0.87 (-1.37, -0.55) | -0.04 (-0.76, 0.44) | 0.08 (-0.58, 0.41) | **0.016** |
| **MELD score** | 123 | 10.0 (8.0, 12.0) | 12.0 (9.0, 16.0) | 13.0 (9.0, 16.0) | 0.2 |
| **History of OHE** | 123 | 0 (0%) | 20 (32%) | 12 (24%) | 0.092 |
| **Sodium (mmol/L)** | 121 | 136.5 (136.0, 137.8) | 136.0 (132.0, 138.0) | 136.0 (133.3, 138.0) | 0.5 |
| **Creatinine (mg/dL)** | 123 | 0.87 (0.82, 1.00) | 1.20 (0.87, 1.66) | 1.37 (0.93, 1.75) | **0.022** |
| **Bilirubin (mg/dL)** | 123 | 0.95 (0.53, 1.50) | 1.11 (0.78, 1.58) | 0.85 (0.58, 1.30) | 0.15 |
| **Cholinesterase (kU/L)** | 115 | 5.31 (4.27, 5.93) | 2.94 (2.42, 4.71) | 2.59 (2.10, 4.25) | **0.005** |
| **AST (U/L)** | 121 | 45 (29, 67) | 38 (31, 48) | 34 (25, 49) | 0.2 |
| **ALT (U/L)** | 120 | 36 (21, 62) | 20 (15, 29) | 19 (12, 30) | **0.044** |
| **Albumin (g/L)** | 120 | 38.0 (34.0, 38.8) | 32.0 (27.8, 36.0) | 30.0 (26.3, 34.0) | **0.029** |
| **CRP (mg/L)** | 120 | 4 (3, 11) | 8 (4, 14) | 10 (6, 29) | 0.086 |
| **WBC (per nL)** | 120 | 4.15 (3.63, 5.58) | 5.45 (3.80, 7.88) | 4.35 (3.40, 7.55) | 0.3 |
| **Hemoglobin (g/dL)** | 120 | 11.90 (9.90, 12.53) | 10.45 (8.85, 12.30) | 9.40 (8.28, 10.55) | **0.011** |
| **Platelets (per nL)** | 120 | 97 (60, 136) | 126 (76, 185) | 114 (77, 175) | 0.5 |
| **INR** | 123 | 1.21 (1.16, 1.37) | 1.21 (1.13, 1.32) | 1.22 (1.14, 1.32) | >0.9 |

## Table S2: Baseline characteristics of patients stratified by center

| **Variable** | **N** | **Hannover**  N = 72 | **Mainz**  N = 51 | **p-value** |
| --- | --- | --- | --- | --- |
| **Age (years)** | 123 | 59 (53, 65) | 60 (51, 65) | >0.9 |
| **Gender** | 123 |  |  | 0.2 |
| Male |  | 46 (64%) | 38 (75%) |  |
| Female |  | 26 (36%) | 13 (25%) |  |
| **Etiology** | 123 |  |  | **0.002** |
| ALD |  | 34 (47%) | 28 (55%) |  |
| MetALD |  | 6 (8.3%) | 1 (2.0%) |  |
| MASLD |  | 5 (6.9%) | 11 (22%) |  |
| Viral |  | 1 (1.4%) | 3 (5.9%) |  |
| Other |  | 11 (15%) | 7 (14%) |  |
| Mixed |  | 15 (21%) | 1 (2.0%) |  |
| **Indication for TIPS** | 123 |  |  | **0.023** |
| Ascites |  | 43 (60%) | 43 (84%) |  |
| Bleeding |  | 17 (24%) | 6 (12%) |  |
| Hydrothorax |  | 1 (1.4%) | 0 (0%) |  |
| Ascites + bleeding |  | 9 (13%) | 1 (2.0%) |  |
| Ascites + hydrothorax |  | 1 (1.4%) | 1 (2.0%) |  |
| Other |  | 1 (1.4%) | 0 (0%) |  |
| **Liver Frailty Index (LFI)** | 123 | 4.35 (3.83, 5.11) | 4.22 (3.73, 4.73) | 0.2 |
| **LFI categorised** | 123 |  |  | 0.6 |
| Robust |  | 5 (6.9%) | 5 (9.8%) |  |
| Pre-frail |  | 35 (49%) | 28 (55%) |  |
| Frail |  | 32 (44%) | 18 (35%) |  |
| **LFI dichotomised** | 123 |  |  | 0.3 |
| Robust/Pre-frail |  | 40 (56%) | 33 (65%) |  |
| Frail |  | 32 (44%) | 18 (35%) |  |
| **CHE (PHES)** | 118 |  |  | 0.5 |
| CHE- |  | 34 (49%) | 27 (55%) |  |
| CHE+ |  | 35 (51%) | 22 (45%) |  |
| **PHES** | 118 | -5.0 (-7.0, -2.0) | -4.0 (-7.0, -1.0) | 0.6 |
| **S-ANT1** | 120 | 22 (18, 29) | 16 (13, 18) | **<0.001** |
| **Child Pugh** | 123 |  |  | 0.2 |
| A |  | 7 (9.7%) | 4 (7.8%) |  |
| B |  | 62 (86%) | 40 (78%) |  |
| C |  | 3 (4.2%) | 7 (14%) |  |
| **FIPS score** | 120 | -0.20 (-1.05, 0.39) | -0.03 (-0.55, 0.55) | 0.13 |
| **MELD score** | 123 | 11.5 (8.0, 15.0) | 13.0 (10.0, 16.0) | 0.077 |
| **History of OHE** | 123 | 20 (28%) | 12 (24%) | 0.6 |
| **Sodium (mmol/L)** | 121 | 135.0 (132.0, 138.8) | 136.0 (134.0, 138.0) | 0.3 |
| **Creatinine (mg/dL)** | 123 | 1.22 (0.87, 1.69) | 1.04 (0.89, 1.59) | 0.7 |
| **Bilirubin (mg/dL)** | 123 | 0.88 (0.53, 1.24) | 1.30 (0.90, 1.80) | **0.001** |
| **Cholinesterase (kU/L)** | 115 | 2.77 (2.16, 4.29) | 3.86 (2.48, 4.94) | **0.021** |
| **AST (U/L)** | 121 | 36 (28, 48) | 38 (30, 53) | 0.5 |
| **ALT (U/L)** | 120 | 22 (15, 34) | 19 (13, 27) | 0.2 |
| **Albumin (g/L)** | 120 | 31.0 (27.0, 36.0) | 31.0 (28.0, 35.0) | >0.9 |
| **CRP (mg/L)** | 120 | 8 (3, 20) | 9 (5, 22) | 0.5 |
| **WBC (per nL)** | 120 | 5.10 (3.60, 7.80) | 5.00 (3.70, 7.80) | 0.7 |
| **Hemoglobin (g/dL)** | 120 | 9.40 (8.25, 11.50) | 10.70 (9.30, 12.30) | **0.009** |
| **Platelets (per nL)** | 120 | 117 (76, 182) | 116 (74, 172) | >0.9 |
| **INR** | 123 | 1.17 (1.11, 1.28) | 1.30 (1.20, 1.45) | **<0.001** |

## Table S3. Univariable Fine and Gray competing risk regression analysis for the association of variables with death under consideration of liver transplantation as competing event.

| **Variable** | **N** | **sHR** | **95% CI** | **p-value** |
| --- | --- | --- | --- | --- |
| Age (y) | 123 | 1.05 | 1.01, 1.10 | 0.015 |
| CHE (PHES) |  |  |  |  |
| No | 61 | — | — |  |
| yes | 57 | 1.89 | 0.84, 4.28 | 0.13 |
| PHES | 118 | 0.94 | 0.85, 1.03 | 0.2 |
| ANT | 120 | 0.97 | 0.92, 1.02 | 0.3 |
| LFI (cont.) | 123 | 1.53 | 1.11, 2.11 | 0.010 |
| LFI (cat.) |  |  |  |  |
| robust | 10 |  |  |  |
| pre-frail | 63 | n/a^a^ |  |  |
| Frail | 50 | n/a^a^ |  |  |
| LFI (cat.) |  |  |  |  |
| robust/pre-frail | 73 | — | — |  |
| Frail | 50 | 1.77 | 0.80, 3.89 | 0.2 |
| LFI (cat.) |  |  |  |  |
| Q1 | 31 | — | — |  |
| Q2-Q4 | 92 | 10.2 | 1.44, 72.0 | 0.020 |
| Gender |  |  |  |  |
| Male | 84 | — | — |  |
| female | 39 | 0.46 | 0.16, 1.33 | 0.2 |
| Indication for TIPS |  |  |  |  |
| Ascites | 86 | — | — |  |
| Bleeding | 23 | 1.06 | 0.41, 2.76 | >0.9 |
| PSG Delta (%) | 123 | 0.28 | 0.03, 2.76 | 0.3 |
| TIPS diameter (mm) | 122 | 0.58 | 0.40, 0.83 | 0.003 |
| Child-Pugh grade |  |  |  |  |
| A | 11 |  |  |  |
| B | 102 | n/a^b^ |  |  |
| C | 10 | n/a^b^ |  |  |
| FIPS score | 120 | 2.17 | 1.35, 3.47 | 0.001 |
| MELD score | 123 | 1.09 | 1.00, 1.19 | 0.054 |
| History of OHE |  |  |  |  |
| No | 91 | — | — |  |
| yes | 32 | 1.89 | 0.83, 4.28 | 0.13 |
| Sodium (mmol/L) | 121 | 0.94 | 0.87, 1.01 | 0.074 |
| Creatinine (mg/dL) | 123 | 1.82 | 1.15, 2.87 | 0.010 |
| Bilirubin (mg/dL) | 123 | 0.92 | 0.43, 1.99 | 0.8 |
| Cholinesterase (kU/L) | 115 | 0.61 | 0.44, 0.85 | 0.003 |
| AST (U/L) | 121 | 1.00 | 0.98, 1.02 | >0.9 |
| ALT (U/L) | 120 | 1.00 | 0.99, 1.01 | 0.8 |
| Albumin (g/L) | 120 | 0.91 | 0.85, 0.96 | 0.002 |
| CRP (mg/L) | 120 | 1.02 | 1.00, 1.03 | 0.016 |
| WBC (per nL) | 120 | 0.97 | 0.87, 1.09 | 0.6 |
| Hemoglobin (g/dL) | 120 | 0.72 | 0.61, 0.86 | <0.001 |
| Platelets (per nL) | 120 | 1.00 | 1.00, 1.00 | 0.9 |
| INR | 123 | 1.68 | 0.21, 13.3 | 0.6 |

^a^ no patient classified as robust died during follow-up.

^b^ no patient with Child A died during follow-up.

## Table S4. Univariable Cox models for the association of variables with transplantation-free survival.

| **Variable** | **N** | **HR** | **95% CI** | **p-value** |
| --- | --- | --- | --- | --- |
| Age (y) | 123 | 1.04 | 1.00, 1.08 | 0.030 |
| CHE (PHES) |  |  |  |  |
| No | 61 | — | — |  |
| yes | 57 | 1.58 | 0.76, 3.32 | 0.2 |
| PHES | 118 | 0.94 | 0.86, 1.03 | 0.2 |
| ANT | 120 | 0.96 | 0.91, 1.01 | 0.10 |
| LFI (cont.) | 123 | 1.41 | 0.97, 2.05 | 0.072 |
| LFI (cat.) |  |  |  |  |
| robust | 10 |  |  |  |
| pre-frail | 63 | n/a^a^ |  |  |
| frail | 50 | n/a^a^ |  |  |
| LFI (cat.) |  |  |  |  |
| robust/pre-frail | 73 | — | — |  |
| frail | 50 | 1.44 | 0.70, 2.95 | 0.3 |
| LFI (cat.) |  |  |  |  |
| Q1 | 31 | — | — |  |
| Q2-Q4 | 92 | 6.22 | 1.48, 26.2 | 0.013 |
| Gender |  |  |  |  |
| male | 84 | — | — |  |
| female | 39 | 0.33 | 0.12, 0.96 | 0.041 |
| Indication |  |  |  |  |
| Ascites | 86 | — | — |  |
| Bleeding | 23 | 0.73 | 0.28, 1.93 | 0.5 |
| PSG Delta (%) | 123 | 0.34 | 0.04, 2.75 | 0.3 |
| TIPS diameter (mm) | 122 | 0.80 | 0.60, 1.05 | 0.10 |
| Child-Pugh grade |  |  |  |  |
| A | 11 |  |  |  |
| B | 102 | n/a^b^ |  |  |
| C | 10 | n/a^b^ |  |  |
| FIPS score | 120 | 2.65 | 1.61, 4.38 | <0.001 |
| MELD score | 123 | 1.14 | 1.06, 1.24 | <0.001 |
| History of OHE |  |  |  |  |
| No | 91 | — | — |  |
| yes | 32 | 2.18 | 1.04, 4.53 | 0.038 |
| Sodium (mmol/L) | 121 | 0.92 | 0.86, 0.99 | 0.021 |
| Creatinine (mg/dL) | 123 | 1.76 | 1.20, 2.58 | 0.004 |
| Bilirubin (mg/dL) | 123 | 1.34 | 0.91, 1.97 | 0.14 |
| Cholinesterase (kU/L) | 115 | 0.58 | 0.43, 0.79 | <0.001 |
| AST (U/L) | 121 | 1.00 | 0.99, 1.01 | 0.8 |
| ALT (U/L) | 120 | 1.00 | 0.99, 1.01 | >0.9 |
| Albumin (g/L) | 120 | 0.92 | 0.87, 0.98 | 0.013 |
| CRP (mg/L) | 120 | 1.02 | 1.00, 1.03 | 0.023 |
| WBC (per nL) | 120 | 0.94 | 0.83, 1.06 | 0.3 |
| Hemoglobin (g/dL) | 120 | 0.80 | 0.67, 0.96 | 0.018 |
| Platelets (per nL) | 120 | 1.00 | 0.99, 1.00 | 0.4 |
| INR | 123 | 6.37 | 1.38, 29.3 | 0.017 |

^a^ no patient classified as robust died during follow-up.

^b^ no patient with Child A died during follow-up.

## Table S5. Multivariable Cox regression analysis for Transplantation-free survival in the subgroup of patients with ascites as indication for TIPS

| **Variable** | **N** | **HR** | **95% CI** | **p-value** |
| --- | --- | --- | --- | --- |
| LFI (cont.) | 87 | 1.00 | 0.63, 1.59 | >0.9 |
| FIPS score | 87 | 1.97 | 1.14, 3.40 | 0.015 |
| LFI (cat.) |  |  |  |  |
| Robust | 4 | — | — |  |
| Pre-frail | 48 | 21,172,848 | 0.00, Inf | >0.9 |
| Frail | 35 | 17,043,612 | 0.00, Inf | >0.9 |
| FIPS score | 87 | 1.92 | 1.12, 3.28 | 0.017 |
| LFI (cat.) |  |  |  |  |
| Q1 | 19 | — | — |  |
| Q2-Q4 | 68 | 3.29 | 0.77, 14.1 | 0.11 |
| FIPS score | 87 | 1.91 | 1.11, 3.27 | 0.019 |

## Table S6. Multivariable Fine and Gray competing risk regression analysis for the development of OHE in the subgroup of patients with ascites as indication for TIPS

| **Variable** | **N** | **sHR** | **95% CI** | **p-value** |
| --- | --- | --- | --- | --- |
| LFI (cont.) | 87 | 0.83 | 0.54, 1.28 | 0.4 |
| PSG delta (%) | 87 | 22.7 | 3.93, 131 | <0.001 |
| FIPS score | 87 | 2.01 | 1.24, 3.25 | 0.005 |
|  |  |  |  |  |
| LFI (cat.) |  |  |  |  |
| Robust | 4 | — | — |  |
| Pre-frail | 48 | 0.62 | 0.05, 7.50 | 0.7 |
| Frail | 35 | 0.48 | 0.04, 6.38 | 0.6 |
| PSG delta (%) | 87 | 21.0 | 3.43, 129 | 0.001 |
| FIPS score | 87 | 1.99 | 1.19, 3.34 | 0.009 |
|  |  |  |  |  |
| LFI (cat.) |  |  |  |  |
| Q1 | 19 | — | — |  |
| Q2-Q4 | 68 | 1.42 | 0.56, 3.59 | 0.5 |
| PSG delta (%) | 87 | 24.0 | 2.83, 203 | 0.004 |
| FIPS score | 87 | 1.84 | 1.16, 2.91 | 0.010 |

## Table S7. Univariable Fine and Gray competing risk regression analysis for the development of OHE

| **Variable** | **N** | **sHR** | **95% CI** | **p-value** |
| --- | --- | --- | --- | --- |
| Age (y) | 123 | 1.03 | 1.00, 1.06 | 0.030 |
| CHE (PHES) |  |  |  |  |
| No | 61 | — | — |  |
| yes | 57 | 1.81 | 0.95, 3.44 | 0.070 |
| PHES | 118 | 0.94 | 0.87, 1.01 | 0.10 |
| ANT | 120 | 0.99 | 0.96, 1.03 | 0.8 |
| LFI (cont.) | 123 | 0.90 | 0.66, 1.24 | 0.5 |
| LFI (cat.) |  |  |  |  |
| robust | 10 | — | — |  |
| pre-frail | 63 | 0.82 | 0.28, 2.37 | 0.7 |
| frail | 50 | 0.69 | 0.23, 2.08 | 0.5 |
| LFI (cat.) |  |  |  |  |
| robust/pre-frail | 73 | — | — |  |
| frail | 50 | 0.82 | 0.44, 1.55 | 0.5 |
| LFI (cat.) |  |  |  |  |
| Q1 | 31 | — | — |  |
| Q2-Q4 | 92 | 0.92 | 0.47, 1.80 | 0.8 |
| Gender |  |  |  |  |
| male | 84 | — | — |  |
| female | 39 | 0.63 | 0.30, 1.34 | 0.2 |
| Indication for TIPS |  |  |  |  |
| Ascites | 86 | — | — |  |
| Bleeding | 23 | 1.51 | 0.74, 3.06 | 0.3 |
| PSG Delta (%) | 123 | 7.21 | 1.39, 37.3 | 0.019 |
| TIPS diameter (mm) | 122 | 1.10 | 0.88, 1.37 | 0.4 |
| Child-Pugh grade |  |  |  |  |
| A | 11 | — | — |  |
| B | 102 | 0.43 | 0.19, 0.98 | 0.044 |
| C | 10 | 0.61 | 0.18, 2.09 | 0.4 |
| FIPS score | 120 | 1.61 | 1.13, 2.28 | 0.008 |
| MELD score | 123 | 1.09 | 1.02, 1.16 | 0.009 |
| History of OHE |  |  |  |  |
| No | 91 | — | — |  |
| yes | 32 | 1.34 | 0.68, 2.65 | 0.4 |
| Sodium (mmol/L) | 121 | 0.96 | 0.91, 1.02 | 0.2 |
| Creatinine (mg/dL) | 123 | 1.52 | 1.05, 2.22 | 0.029 |
| Bilirubin (mg/dL) | 123 | 1.03 | 0.76, 1.38 | 0.9 |
| Cholinesterase (kU/L) | 115 | 0.95 | 0.79, 1.14 | 0.6 |
| AST (U/L) | 121 | 0.99 | 0.98, 1.00 | 0.12 |
| ALT (U/L) | 120 | 0.99 | 0.98, 1.01 | 0.3 |
| Albumin (g/L) | 120 | 1.02 | 0.96, 1.08 | 0.5 |
| CRP (mg/L) | 120 | 1.01 | 0.99, 1.03 | 0.3 |
| WBC (per nL) | 120 | 0.94 | 0.84, 1.05 | 0.3 |
| Hemoglobin (g/dL) | 120 | 0.84 | 0.73, 0.98 | 0.022 |
| Platelets (per nL) | 120 | 0.99 | 0.99, 1.00 | 0.003 |
| INR | 123 | 5.53 | 1.58, 19.3 | 0.007 |

## Table S8. Univariable Fine and Gray competing risk regression analysis for the development of OHE (subtests of the LFI)

| **Variable** | **N** | **sHR** | **95% CI** | **p-value** |
| --- | --- | --- | --- | --- |
| Hand grip strength (kg) | 123 | 1.01 | 0.99, 1.04 | 0.4 |
| Chair stands (s) | 123 | 1.00 | 0.98, 1.02 | >0.9 |
| Position: Side-by-side (s) | 123 | 1.04 | 0.90, 1.20 | 0.6 |
| Position: Semi-Tandem (s) | 123 | 1.08 | 0.92, 1.25 | 0.4 |
| Position: Tandem | 123 | 1.08 | 0.98, 1.19 | 0.12 |

## Table S9: Baseline characteristics of patients with available LFI data at 0, 1, 3, and 6 months (n = 41)

| **Variable** | **N** | **N = 41** |
| --- | --- | --- |
| **Age (years)** | 41 | 56 (51, 64) |
| **Gender** | 41 |  |
| male |  | 32 (78%) |
| female |  | 9 (22%) |
| **Etiology** | 41 |  |
| ALD |  | 22 (54%) |
| MetALD |  | 1 (2.4%) |
| MASLD |  | 4 (9.8%) |
| Viral |  | 2 (4.9%) |
| Other |  | 6 (15%) |
| Mixed |  | 6 (15%) |
| **Indication for TIPS** | 41 |  |
| Ascites |  | 26 (63%) |
| Bleeding |  | 9 (22%) |
| Hydrothorax |  | 0 (0%) |
| Ascites + bleeding |  | 5 (12%) |
| Ascites + hydrothorax |  | 1 (2.4%) |
| Other |  | 0 (0%) |
| **Liver Frailty Index (LFI)** | 41 | 4.22 (3.67, 4.71) |
| **LFI categorised** | 41 |  |
| Robust |  | 6 (15%) |
| Pre-frail |  | 19 (46%) |
| Frail |  | 16 (39%) |
| **CHE (PHES)** | 39 |  |
| CHE- |  | 19 (49%) |
| CHE+ |  | 20 (51%) |
| **PHES** | 39 | -5.0 (-8.0, -2.0) |
| **S-ANT1** | 40 | 19 (14, 22) |
| **Child Pugh** | 41 |  |
| A |  | 7 (17%) |
| B |  | 33 (80%) |
| C |  | 1 (2.4%) |
| **FIPS score** | 39 | -0.58 (-1.07, -0.05) |
| **MELD score** | 41 | 11.0 (8.0, 14.0) |
| **History of OHE** | 41 | 7 (17%) |
| **Sodium (mmol/L)** | 40 | 136.5 (132.8, 139.0) |
| **Creatinine (mg/dL)** | 41 | 0.98 (0.81, 1.55) |
| **Bilirubin (mg/dL)** | 41 | 0.90 (0.58, 1.30) |
| **Cholinesterase (kU/L)** | 37 | 3.76 (2.52, 5.36) |
| **AST (U/L)** | 40 | 40 (31, 53) |
| **ALT (U/L)** | 39 | 22 (15, 40) |
| **Albumin (g/L)** | 39 | 34.0 (28.0, 36.5) |
| **CRP (mg/L)** | 39 | 8 (3, 24) |
| **WBC (per nL)** | 40 | 4.50 (3.70, 6.85) |
| **Hemoglobin (g/dL)** | 40 | 10.90 (9.00, 12.30) |
| **Platelets (per nL)** | 40 | 124 (72, 191) |
| **INR** | 41 | 1.20 (1.12, 1.30) |

## Table S10: Baseline characteristics of patients with available LFI data at 0 and 6 months (n = 52)

| **Variable** | **N** | **N = 52** |
| --- | --- | --- |
| **Age (years)** | 52 | 56 (51, 64) |
| **Gender** | 52 |  |
| male |  | 38 (73%) |
| female |  | 14 (27%) |
| **Etiology** | 52 |  |
| ALD |  | 27 (52%) |
| MetALD |  | 3 (5.8%) |
| MASLD |  | 5 (9.6%) |
| Viral |  | 2 (3.8%) |
| Other |  | 7 (13%) |
| Mixed |  | 8 (15%) |
| **Indication for TIPS** | 52 |  |
| Ascites |  | 35 (67%) |
| Bleeding |  | 10 (19%) |
| Hydrothorax |  | 0 (0%) |
| Ascites + bleeding |  | 6 (12%) |
| Ascites + hydrothorax |  | 1 (1.9%) |
| Other |  | 0 (0%) |
| **Liver Frailty Index (LFI)** | 52 | 4.18 (3.67, 4.71) |
| **LFI categorised** | 52 |  |
| Robust |  | 7 (13%) |
| Pre-frail |  | 26 (50%) |
| Frail |  | 19 (37%) |
| **CHE (PHES)** | 49 |  |
| CHE- |  | 24 (49%) |
| CHE+ |  | 25 (51%) |
| **PHES** | 49 | -5.0 (-7.0, -2.0) |
| **S-ANT1** | 51 | 20 (15, 24) |
| **Child Pugh** | 52 |  |
| A |  | 7 (13%) |
| B |  | 43 (83%) |
| C |  | 2 (3.8%) |
| **FIPS score** | 50 | -0.61 (-1.12, 0.06) |
| **MELD score** | 52 | 10.0 (8.0, 14.0) |
| **History of OHE** | 52 | 9 (17%) |
| **Sodium (mmol/L)** | 51 | 136.0 (132.5, 138.0) |
| **Creatinine (mg/dL)** | 52 | 0.98 (0.81, 1.56) |
| **Bilirubin (mg/dL)** | 52 | 0.92 (0.52, 1.35) |
| **Cholinesterase (kU/L)** | 47 | 3.49 (2.51, 5.11) |
| **AST (U/L)** | 51 | 39 (30, 55) |
| **ALT (U/L)** | 50 | 21 (15, 36) |
| **Albumin (g/L)** | 50 | 32.0 (27.0, 35.8) |
| **CRP (mg/L)** | 50 | 8 (3, 21) |
| **WBC (per nL)** | 51 | 5.1 (3.8, 7.5) |
| **Hemoglobin (g/dL)** | 51 | 10.40 (9.10, 12.15) |
| **Platelets (per nL)** | 51 | 130 (75, 194) |
| **INR** | 52 | 1.20 (1.12, 1.30) |
|  |  |  |
